# Heat Modulation on Target Thermal Bath via Coherent Auxiliary Bath

**DOI:** 10.3390/e23091183

**Published:** 2021-09-08

**Authors:** Wen-Li Yu, Tao Li, Hai Li, Yun Zhang, Jian Zou, Ying-Dan Wang

**Affiliations:** 1 School of Computer Science and Technology, Shandong Technology and Business University, Yantai 264000, China; yuwl@sdtbu.edu.cn; 2 School of Information and Electronic Engineering, Shandong Technology and Business University, Yantai 264000, China; 2018420125@sdtbu.edu.cn (T.L.); 2018420092@sdtbu.edu.cn (Y.Z.); 3 School of Physics, Beijing Institute of Technology, Beijing 100081, China; 4 Institute of Theoretical Physics, Chinese Academy of Sciences, Beijing 100190, China; yingdan.wang@itp.ac.cn; 5 School of Physical Sciences, University of Chinese Academy of Sciences, No. 19A Yuquan Road, Beijing 100049, China; 6 Synergetic Innovation Center for Quantum Effects and Applications, Hunan Normal University, Changsha 410081, China; 7 CAS Center for Excellence in Topological Quantum Computation, University of Chinese Academy of Sciences, Beijing 100190, China

**Keywords:** heat modulation, multifunctional thermal device, coherent auxiliary bath, heat current

## Abstract

We study a scheme of thermal management where a three-qubit system assisted with a coherent auxiliary bath (CAB) is employed to implement heat management on a target thermal bath (TTB). We consider the CAB/TTB being ensemble of coherent/thermal two-level atoms (TLAs), and within the framework of collision model investigate the characteristics of steady heat current (also called target heat current (THC)) between the system and the TTB. It demonstrates that with the help of the quantum coherence of ancillae the magnitude and direction of heat current can be controlled only by adjusting the coupling strength of system-CAB. Meanwhile, we also show that the influences of quantum coherence of ancillae on the heat current strongly depend on the coupling strength of system—CAB, and the THC becomes positively/negatively correlated with the coherence magnitude of ancillae when the coupling strength below/over some critical value. Besides, the system with the CAB could serve as a multifunctional device integrating the thermal functions of heat amplifier, suppressor, switcher and refrigerator, while with thermal auxiliary bath it can only work as a thermal suppressor. Our work provides a new perspective for the design of multifunctional thermal device utilizing the resource of quantum coherence from the CAB.

## 1. Introduction

Quantum thermodynamics mainly studies thermodynamic behaviors emerging in systems that are quantum in nature [1,2,3,4]. Compared with the classical systems dominated by the standard laws of thermodynamics, some novel phenomena can emerge in the quantum systems due to the presence of quantum properties, e.g., quantum coherence [5,6,7,8,9], or entanglement [10,11,12]. For example, the efficiency beyond the Carnot cycle [13], the reversion of heat flowing from the hot system to the colder one [14]. These non-intuitive physical behaviors have become an apparent challenge to the standard laws of thermodynamics [15,16]. The rapid progress of quantum technologies has allowed us to characterize the quantum machine [17,18,19,20] and experimentally realized them in various quantum systems [21,22,23,24,25,26,27,28,29,30]. With the aid of these controllable quantum platforms (systems), some studies focus on the redefinitions of some concepts, such as work and heat [31,32,33,34], and the verification and modification of thermodynamics second law [35] in quantum domain. Others concentrate on heat control/management [36,37,38,39,40,41,42,43] in order to design the quantum thermodynamic process (or quantum thermal machines) that can implement a certain task (or multi-task) using thermal resources [44,45,46,47,48,49,50,51,52,53,54,55,56,57,58,59].

Analog to electronic devices of diode and transistors perfectly regulating the electricity in circuit, it is interesting to investigate whether the heat transferring in the quantum system can also be regulated well like the electric current. Some previous studies have been devoted to thermal rectification [60,61,62]. Currently, the quantum thermal transistor [39] to implement heat amplification, and various quantum control devices of heat current with a specific function have been proposed, such as quantum thermal diodes [47,63], quantum heat switch [64], transistors [48,49], thermometers [50,51,52,53], thermal valves [54] and many-body quantum thermal rectification [65]. Most currently, to design a multifunctional quantum thermal device [20,55,56,57,58,59,66], i.e., integrating the multiple functions into a single device, has become an interesting and active subject. Based on some simple quantum systems, such as a qutrit [58], two coupled qubits [66] and three qubits [39,59] the multifunctional devices have been designed, and under the suitable selections of system’s structure and dynamic parameters these thermal devices can implement two or more functions of amplifier, modulator, switcher, valve, stabilizer, and rectifier. These studies further enrich the applications of quantum small systems. However, it is noted that the multifunctional devices designed in previous investigations have a similar structure of electronic-like transistor where three independent thermal baths with different temperatures are connected to the quantum device, and many dynamic parameters are usually involved to carry out the thermal control of a certain function. In addition, the quantum effects influencing the dynamics of thermal system, such as quantum coherence or entanglement completely come from the system itself due to the coherent interactions among subsystems [20,67]. Therefore, of particular interest to us is whether a quantum device can operate as a multifunctional thermal machine with two baths, and execute the flexible switching among multiple functions by fewer controllable parameters or even a single one. Besides, although plenty of studies have contributed to the roles of quantum coherence in work extraction [68,69], heat to work conversion [70,71], thermal transfer [72] and information scrambling, [73] etc. The effects of quantum coherence outside the system on heat management (or design for a multifunctional thermal device) have not been addressed.

Motivated by the quests above, we, in this paper, design a thermal management scheme where a three-qubit system assisted with a coherent auxiliary bath (CAB) can work as a multifunctional thermal device to implement the heat management on a given thermal bath called target thermal bath (TTB), i.e., to control the magnitude and the direction of heat current flowing into/out of the TTB. We, in the framework of collision model (or repeated interaction model), will investigate the behaviors of steady heat current between the system and the TTB (also named target heat current—THC, hereafter) and thermal functions of the system with a CAB. Here, it is pointed out that the collision model has become a convenient and powerful tool for studying the dynamics of open quantum system [74], especially for the situations of non-equilibrium bath with quantum effects [75,76,77]. Thus, so far, the general thermodynamic framework of collision models has been explored deeply and established [35,78,79,80]. Especially, the multipartite collision models protocol [79,81] might provide a promising way in the design of low-dimensional solid-state thermal management devices. Here, it is an interesting question to integrating the multiple functions into a multipartite device to perform the heat management with the help of quantum effects from nonequilibrium bath. In this work, within the framework of collision models we construct a scheme of thermal management of a three-qubit associated with a nonequilibrium CAB. We want to investigate whether the heat current can be controlled effectively with the help of the CAB connected to one side of system. The results show that the quantum coherence in ancillary bath indeed serves as a resource utilized to control the THC for the amplification, suppression or even reverse of THC. Meanwhile, we find that the effects of quantum coherence including the coherence magnitude and relative phase of ancillae strongly depend on the coupling strength of system—CAB (or system-ancillae). Specifically, the coherence of CAB, for a certain value of coupling strength of system—CAB, seems to be frozen to the THC, i.e., quantum coherence almost has no effects on the THC at the critical value. For the coupling strength below/over the critical value the THC increases/decreases monotonically as the coherence magnitude of ancillae increases. Thus, whether the coherence is positive, negative, or not correlated with the THC strongly depends on the coupling strength of system—CAB. Especially, the reversal-THC, in some parametric regime of the coherence of ancillae and the coupling strength of system-CAB, could appear which is impossible for the auxiliary bath being thermal bath without coherence. Therefore, with the aid of CAB the three-qubit system can serve as a multifunctional thermal device integrating the functions of heat amplifier, suppressor, switcher and refrigerator. Of particular interest, the multiple functions can be switched only by adjusting a single controllable coupling strength of system—CAB.

The remainder of this paper is arranged as follows: In Section 2, we introduce the scheme of heat modulation of three-qubit system as a quantum thermal device and its dynamics. In Section 3, we mainly focus on the effects of ancilla’s coherence (including the relative phase and coherence magnitude) of CAB and coupling strength of system-CAB on the THC, and analyze the thermal functions of system with CAB in different parameter regions. We compare the characteristics of THC in CAB with that in thermal auxiliary bath, and demonstrate the role of coherence of CAB as a resource in thermal modulation. In addition, the effects of temperature on the THC are also discussed. We conclude the whole work in Section 4.

## 2. Model

**Figure 1:**
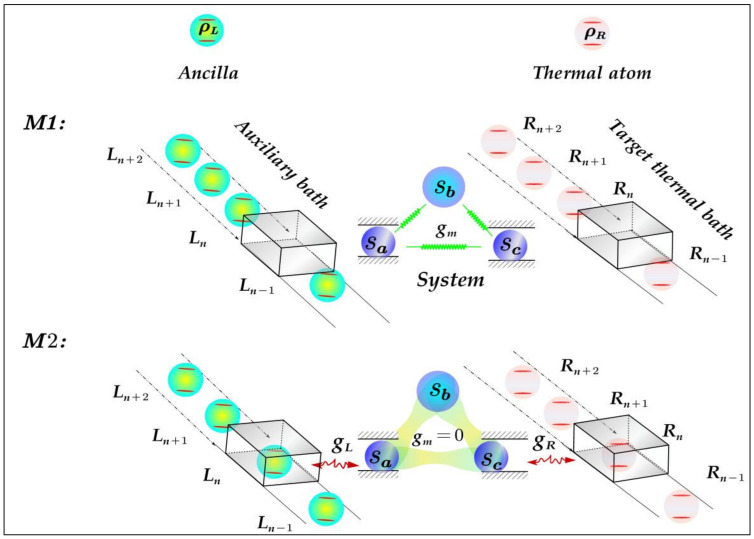
Schematic of heat modulation on the target thermal bath (TTB). The internal interaction of tripartite system ($S_{a,b,c}$ ) is first implemented in M1 process; then the subsystems $S_{a}$ and $S_{c}$ are coupled to the nth ancilla (prepared in $\rho_{L}$) in coherent auxiliary bath (CAB) and the nth thermal atom (prepared in $\rho_{R}$) in TTB, respectively, in M2 process. After that, the two steps of M1 and M2 are implemented repeatedly, and ancilla (thermal atom) in CAB (TTB) interacting with $S_{a}$ ($S_{c}$) are refreshed by the next one in each round. Thus, the steady heat current between the system and TTB, after many rounds, is established.

We design a scheme of heat modulation on a TTB via a tripartite system as quantum machine assisted with an auxiliary bath as shown in Figure 1. In our model, the system is composed of three qubits ($S_{a,b,c}$) with the frequencies $\omega_{a,b,c}$. The CAB (TTB) consists of a series of identical and independent two-level atoms (TLAs) $\left\{L_{1},\;L_{2},\;\ldots \;,\;L_{N}\right\}$ ($\left\{R_{1},\;R_{2},\;\ldots \;,\;R_{N}\right\}$) with transition frequency $\omega_{L}$ ($\omega_{R}$) and density matrix $\rho_{L}$ ($\rho_{R}$). We assume that the ancillae of left auxiliary bath and the thermal TLAs of right TTB synchronously pass through the left box and the right box one by one, respectively, and two processes denoted as M1 (interaction process among three subsystems) and M2 (interaction process between the system and the baths) are implemented alternatively with equal time interval τ. Here, the boxes simulate triggers to control which interaction channels are on/off in the dynamics of system. Specifically, before the atoms reach the boxes, i.e., in M1 process, there are only interactions among the three qubits ($S_{a,b,c}$). Further, when the atoms entering the boxes, i.e., in M2 process, the interaction channels in M1 are off, and the subsystems $S_{a}$($S_{c}$) couples to the ancillae (thermal TLA) in the left (right) box. The energy exchange among the CAB, system and TTB occurs in this process. After the repeated implementations of M1 and M2 processes, a steady heat current between the system and the TTB can be established. With the aid of this model, the influences of coherence of CAB on THC and the thermal functions of system assisted with CAB can be exploited at length.

Next, we focus on the specific dynamics of system in a single round (i.e., to implement the M1 and M2 process once). Here, we denote ${\hat{H}}_{i}=\hbar \omega_{i}{\hat{\sigma }}_{i}^{z}/2$, (i=L, R, a, b and c) as the free Hamiltonians of ancillae in CAB and thermal TLAs in TTB and subsystems $S_{a}$, $S_{b}$ and $S_{c}$, respectively, where $\omega_{i}$ and ${\hat{\sigma }}_{i}^{z}=|1\rangle \langle 1|-|0\rangle \langle 0|$ are independently the transition frequencies and Pauli matrices of the TLAs with subscript i, and $|1\rangle$ ($|0\rangle$) describing a TLA in the excited (ground) state. The free Hamilton of system is denoted as ${\hat{H}}_{0}={\hat{H}}_{a}+{\hat{H}}_{b}+{\hat{H}}_{c}$. In M1 process, the interaction Hamilton of system is given as:

(1) $${\hat{V}}_{1}=g_{m}{\hat{\sigma }}_{a}^{x}⊗{\hat{\sigma }}_{b}^{x}⊗{\hat{\sigma }}_{c}^{x}={\hat{V}}_{R}+{\hat{V}}_{OR}$$

with $g_{m}$ is the coupling constant among three subsystems. ${\hat{V}}_{OR}=g_{m}({\hat{\sigma }}_{a}^{+}{\hat{\sigma }}_{b}^{+}{\hat{\sigma }}_{c}^{+}+$ ${\hat{\sigma }}_{a}^{-}{\hat{\sigma }}_{b}^{-}{\hat{\sigma }}_{c}^{-}+{\hat{\sigma }}_{a}^{+}{\hat{\sigma }}_{b}^{+}{\hat{\sigma }}_{c}^{-}+{\hat{\sigma }}_{a}^{-}{\hat{\sigma }}_{b}^{-}{\hat{\sigma }}_{c}^{+}+{\hat{\sigma }}_{a}^{+}{\hat{\sigma }}_{b}^{-}{\hat{\sigma }}_{c}^{-}+{\hat{\sigma }}_{a}^{-}{\hat{\sigma }}_{b}^{+}{\hat{\sigma }}_{c}^{+})$ and ${\hat{V}}_{R}=g_{m}({\hat{\sigma }}_{a}^{+}{\hat{\sigma }}_{b}^{-}{\hat{\sigma }}_{c}^{+}+{\hat{\sigma }}_{a}^{-}{\hat{\sigma }}_{b}^{+}{\hat{\sigma }}_{c}^{-})$ (${\hat{\sigma }}_{i}^{+}=\;|1\rangle \langle 0|$ (${\hat{\sigma }}_{i}^{-}=|0\rangle \langle 1|$) being the raising (lowering) operator) representing the off-resonant and the resonant terms, respectively. Here, the *XXX*-type Hamiltonian as a more likely candidate of three-qubit physical Hamiltonian [82] has been exploited widely in spin system [82,83,84,85], and can be implemented experimentally [59]. Further, its dynamics effectively reduces to that of the resonant coupling ${\hat{V}}_{R}$ on phenomenological grounds [86,87] when the frequencies are resonant $\omega_{b}=\omega_{a}+\omega_{c}$ and the coupling is weak $g_{m}\ll \omega_{i}$ i.e., the rotating wave approximation applies (the off-resonant term ${\hat{V}}_{OR}$ in the interaction Hamiltonian (1) can be omitted). In this paper, we also consider that the condition of resonant frequencies is satisfied. According to the scheme in Figure 1, the dynamics of the system in M1 process for $t\in \;\left(t_{n-1},\;t_{n-1}+\tau \right]$ is given as:

(2) $$M1:{\;\rho }_{S}^{n}:=\Lambda_{1}({\tilde{\rho }}_{S}^{n-1})={\hat{U}}_{1}(\tau ){\tilde{\rho }}_{S}^{n-1}{\hat{U}}_{1}^{+}(\tau ),$$

where the mapping $\Lambda_{1}$ describes the unitary evolution of system, and the unitary operator is ${\hat{U}}_{1}(\tau )=\exp(-iH_{S}\tau /\hbar )$ with ${\hat{H}}_{S}={\hat{H}}_{0}+{\hat{V}}_{1}$ being the total Hamilton of system. ${\tilde{\rho }}_{S}^{n-1}$ ($\rho_{S}^{n}$) represents the state of system S before (after) the (n−1)th ancilla reaches (leaves) the box, that is, the state of beginning (ending) of system’s internal interaction at time $t_{n-1}$ ($t_{n-1}+\tau$). One can identify that the free Hamilton of ${\hat{H}}_{0}$ does not commute with the unitary operator ${\hat{U}}_{1}(\tau )$ for the finite coupling $g_{m}$, i.e., $\left[{\hat{H}}_{0},{\hat{U}}_{1}(\tau )\right]\neq 0$ which implies that the energy of system is non-preserving in this process due to the off-resonant term ${\hat{V}}_{OR}$ introduced. Here, the off-resonant term ${\hat{V}}_{OR}$ characterizes the external driven on the system by an external agent (or work source). Any energy changes of system in M1 process are solely due to energy leaving or entering the work external agent. In the M2 process for $t\in \;\left(t_{n-1}+\tau ,\;t_{n}\right]$ with $t_{n}=t_{n-1}+2\tau$ (i.e., at the time interval of the nth ancillae (TLA in TTB) kept in the left (right) box), the interaction channel in M1 process is switched-off, and the subsystems $S_{a}$ and $S_{c}$ are coupled to the ancillae in left box and the thermal TLA in right box, respectively. The interactions are expressed as:

(3) $${\hat{V}}_{S,L(R)}=g_{L(R)}({\hat{\sigma }}_{L(R)}^{x}{\hat{\sigma }}_{a(c)}^{x}+{\hat{\sigma }}_{L(R)}^{y}{\hat{\sigma }}_{a(c)}^{y}+{\hat{\sigma }}_{L(R)}^{z}{\hat{\sigma }}_{a(c)}^{z}),$$

where ${\hat{V}}_{S,L}$ (${\hat{V}}_{S,R}$) and $g_{L}$ ($g_{R}$) represent the interaction Hamilton and the coupling constant of $S_{a}$- ancillae in the left box ($S_{c}$-TLA in the right box), respectively. The dynamics of system and TTB are unitary and can be described as:

(4) $$M2:{\tilde{\rho }}_{S(R)}^{n}:{=\Lambda }_{2}(\rho_{S(R)}^{n})={\mathrm{tr}}_{L+R(S)}\left[{\hat{U}}_{2}(\tau )\rho_{S}^{n}⊗\rho_{L}^{n}⊗\rho_{R}^{n}{\hat{U}}_{2}^{+}(\tau )\right],$$

where ${\tilde{\rho }}_{S}^{n}$ (${\tilde{\rho }}_{R}^{n}$) denotes the state of the system (nth thermal TLA of TTB) at time $t_{n}=2n\tau$ (i.e., the moment of the nth thermal TLA in TTB just leaving the right box) the mapping $\Lambda_{2}$ corresponds to a Markov process and ${\hat{U}}_{2}(\tau )={\hat{U}}_{S,L}(\tau ){\hat{U}}_{S,R}(\tau )$ with:

(5) $${\hat{U}}_{S,L(R)}(\tau )=\exp\left[-iV_{S,L(R)}\tau /\hbar \right].$$

Here, it is noted that as usually treated in most works of collision models [35,65,70,71,72,73] we have considered that the state of system after its interaction with two baths is embodied by the stroboscopic map. That ensures the system is being always independent to the particle units (ancillae/thermal-TLAs in CAB/TTB) that have collided, and the dynamics of system a memoryless Markov process. Physically, the collision model protocol is consistent with the inspiration from Boltzmann’s original Stosszahlansatz. For instance, a particle in Brownian motion interacts with only a few water molecules at a time. Moreover, this interaction lasts for an extremely short time, after that the molecule moves on, and never to return [88]. Since the environment is large, the decoherence and dissipation ensure that the next molecule to arrive will be completely uncorrelated from the previous one, so the process repeats anew [35]. The same scenario is also suitable for the multipartite collision models [79,81], and addressing the thermodynamics of engineered reservoirs [37,89,90,91]. In terms 89 of the exchange interaction of ${\hat{V}}_{S,L(R)}$ given in Equation (3) one can identify that the commutation relation $\left[{\hat{H}}_{S,L(R)}^{0},{\hat{U}}_{S,L(R)}(\tau )\right]=0$ with ${\hat{H}}_{S,L}^{0}={\hat{H}}_{a}+{\hat{H}}_{L}$ and ${\hat{H}}_{S,R}^{0}={\hat{H}}_{c}+{\hat{H}}_{R}$ holds, which indicates that the total energy of whole system (system plus CAB and TTB) is preserved in this process. Here, it is mentioned that the unitary evolution ${\hat{U}}_{S,L(R)}(\tau )$ in Equation (5) above corresponds to a swap gate operation, and we can rewrite it as:

(6) $${\hat{U}}_{S,L(R)}(\tau )=(\cos(2\lambda_{L(R)})){\hat{I}}_{L(R),a(c)}+i(\sin(2\lambda_{L(R)})){\hat{S}}_{L(R),a(c)}^{sw},$$

where $\lambda_{L(R)}{=g}_{L(R)}\tau$ is the dimensionless coupling strength of system-ancillae in auxiliary bath (system-TLAs in TTB), ${\hat{I}}_{L(R),a(c)}$ is the 4×4 identity operator, and ${\hat{S}}_{L(R),a(c)}^{sw}=|11\rangle \langle 11|+|00\rangle \langle 00|+|01\rangle \langle 10|+|10\rangle \langle 01|$ is the two-TLAs swap operator, having ${\hat{S}}_{L(R),a(c)}^{sw}\left(|\psi_{L(R)}\rangle ⊗|\psi_{a(c)}\rangle \right)=|\psi_{a(c)}\rangle ⊗|\psi_{L(R)}\rangle$ for all $|\psi_{a(c)}\rangle ,|\psi_{L(R)}\rangle \in {\mathbb{C}}^{2}$. Therefore, from Equation (6), the swap strength between two particles is determined by $\left|\sin(2\lambda_{L(R)})\right|$ varying with $\lambda_{L(R)}$ at the period π/2. Further, when $\lambda_{L(R)}=k\pi /2\;(k=0,\;1,\;2,\;\ldots \;,\;n)$ the swap strength $\left|\sin(2\lambda_{L(R)})\right|$ is zero conrresponding to the weakest swap strength, and for $\lambda_{L(R)}=k\pi /2+\pi /4$ it becomes one characterizing the strongest swap strength (complete swap) between the system and the ancillae in CAB (thermal atoms in TTB). It also means that the energy exchange between the system and the baths (CAB and TTB) will be periodical with the same period as that of swap strength. In our model, only the heat exchange between the system and the TTB occurs in the M2 process due to the energy-persevering evolution. In the same spirit as the definition of heat (or heat flow) in [35,61,78,92,93], the amount of heat exchange between the system and the TTB can be quantified by the energy change of thermal TLAs of TTB in each round. In the arbitrary n-th round the amount of heat flowing to TTB reads:

(7) $$\Delta Q_{n}=\mathrm{tr}\left[({\tilde{\rho }}_{R}^{n}{-\rho }_{R}^{n}){\hat{H}}_{R}\right],$$

where $\rho_{R}^{n}$ (${\tilde{\rho }}_{R}^{n}$) is the state of the n-th thermal TLA in TTB at the beginning (end) of the subsystem $S_{c}$ in M2 process. Here, $\Delta Q_{n}>0$ ($\Delta Q_{n}<0$) represents that the system pumps heat into the TTB (the TTB delivers heat to the system). It is pointed out that the state $\rho_{R}^{n}$ of the n-th thermal TLA is initially a thermal state and, the state, after the mapping M2, ${\tilde{\rho }}_{R}^{n}$ remains in a diagonal distribution in the eigenbasis of ${\hat{H}}_{R}$ in our model (i.e., both of them are the mixed states with no coherence), and the energy spectrum of ${\hat{H}}_{R}$ is kept unchanged in the process: $\rho_{R}^{n}\to {\tilde{\rho }}_{R}^{n}$. Based on the heat exchange in Equation (7) between the system and the TTB, the average heat current can be defined as:

(8) $$J_{n}:=\frac{{\Delta Q}_{n}}{2\tau }=\mathrm{tr}\left[\frac{1}{2\tau }\left({\tilde{\rho }}_{R}^{n}{-\rho }_{R}^{n}\right){\hat{H}}_{R}\right].$$

The positive (negative) current $J_{n}>0$ ($J_{n}<0$) in Equation (8) indicates the heat flowing into (out of) the TTB, which also means that the system works as a heat pump ($J_{n}>0$) (refrigerator ($J_{n}<0$)) to heat (refrigerate) the TTB. In a long-time limit, the steady THC denoted as $J^{\mathrm{SS}}$ can be established:

(9) $$J^{\mathrm{SS}}=\underset{n\to \infty }{\lim}\mathrm{tr}\left[\frac{1}{2\tau }\left({\tilde{\rho }}_{R}^{n}{-\rho }_{R}^{n}\right){\hat{H}}_{R}\right]=\mathrm{tr}\left[\frac{1}{2\tau }\left({\tilde{\rho }}_{R}^{n+1}{-\rho }_{R}^{n+1}\right){\hat{H}}_{R}\right].$$

It is well known that when a finite system contacts with an infinite heat/nonequilibrium bath it will relax to (or be thermalized into) a steady state as the time increases, and a dynamical equilibrium can be established among the system and the baths, i.e., the steady heat current emerged. This mechanism is also suitable for our collision model. Specifically, the interaction between the three-qubit system and the two baths in each round is used to mimic the thermal contact process in the conventional model, and the state of system, after each round, can be updated once including the populations and quantum correlation/coherence among qubits. As the collision time increases the system’s state is modified by the baths less and less gradually, and the system, after many rounds, will reach a steady state associated with the steady heat current. In the following subsections, we are mainly concerned about the behaviors of THC and the thermal functions of the system with a thermal/coherent auxiliary bath. For the sake of brevity, the THC mentioned in the following subsections refers to the steady THC.

## 3. Modulation of Heat Current via Auxiliary Bath

### 3.1. Initial States of System and Baths

We consider that the CAB, system and TTB are initially uncorrelated, and the initial state of the whole composite system (system plus CAB and TTB) is:

(10) $$\rho^{\mathrm{tot}}\left(0\right)=\rho_{L}^{\mathrm{tot}}\left(0\right)⊗\rho_{S}\left(0\right)⊗\rho_{R}^{\mathrm{tot}}\left(0\right).$$

Here $\rho_{S}\left(0\right)$ is the initial state of system being a thermal product state of three subsystems given as:

(11) $$\rho_{S}\left(0\right)=\underset{i=a,b,c}{⊗}\exp\left({-\beta \hat{H}}_{i}\right)/Z\left({\hat{H}}_{i}\right),$$

where $Z\left(\hat{H}\right)=\mathrm{tr}\left[\exp\left(-\beta \hat{H}\right)\right]$ is the partition function with the inverse temperature $\beta =1{/k}_{B}T$ (set Boltzmann constant $k_{B}=1$). In Equation (10), $\rho_{L}^{\mathrm{tot}}\left(0\right)={⊗}_{j=1}^{N}\rho_{L,R}^{j}$$={\left[\rho_{L,R}\left(0\right)\right]}^{⊗N}$ represent the initial states of CAB ($\rho_{L}^{\mathrm{tot}}\left(0\right)$) and TTB ($\rho_{R}^{\mathrm{tot}}\left(0\right)$) where each bath of CAB and TTB is composed of identical units. Further, the initial state of each thermal TLA $\rho_{R}\left(0\right)$ in TTB and $\rho_{L}\left(0\right)$ for the ancillae in CAB are independently given by:

(12) $$\rho_{R}\left(0\right)=Z^{-1}\left({\hat{H}}_{R}\right)\exp\left({-\beta \hat{H}}_{R}\right),$$

and

(13) $$\rho_{L}\left(0\right)=(1-\alpha )\rho_{\beta }+\alpha |\phi_{L}\rangle \langle \phi_{L}|,$$

where $|\phi_{L}\rangle =\sqrt{P_{00}}|0\rangle +e^{i\theta }\sqrt{P_{11}}|1\rangle$ with θ being the relative phase and $\rho_{\beta }{=Z}^{-1}\left({\hat{H}}_{L}\right)\exp\left({-\beta \hat{H}}_{L}\right)=\sum_{X=0,1}P_{\mathrm{XX}}|X\rangle \langle X|$ with $P_{\mathrm{XX}}=\langle X|Z^{-1}\left({\hat{H}}_{L}\right)\exp\left({-\beta \hat{H}}_{L}\right)|X\rangle$ (X=0, 1); α (0≤α≤1) is a weight parameter determining the proportion of two components, thermal state $\rho_{\beta }$ and pure coherent state $|\phi_{L}\rangle$, in the state $\rho_{L}\left(0\right)$. Here, it is noticed that the parameter α only appears in the non-diagonal elements of $\rho_{L}\left(0\right)$, and the diagonal elements of $\rho_{L}\left(0\right)$ are the same with the ones of thermal state $\rho_{\beta }$. According to the coherence measure of $l_{1}$-norm, for an arbitrary state ρ the coherence reads [5]

(14) $${\mathbb{C}}_{l_{1}}\left(\rho \right)=\sum_{m,n\left(m\neq n\right)}\left|\rho_{\mathrm{mn}}\right|,$$

with $\rho_{\mathrm{mn}}$ (m≠n) the non-diagonal elements of ρ. One can identify that for the state $\rho_{L}\left(0\right)$ given in Equation (13) the $l_{1}$-measure of coherence ${\mathbb{C}}_{l_{1}}\left(\rho_{L}\left(0\right)\right)=2\alpha \sqrt{P_{00}P_{11}}$ is proportional to α. That is, when fixing the population $P_{00}$ ($P_{11}=1-P_{00}$) of $\rho_{L}\left(0\right)$ in the energy basis $\left\{|0\rangle ,|1\rangle \right\}$ of ${\hat{H}}_{L}$ the coherence ${\mathbb{C}}_{l_{1}}\left(\rho_{L}\left(0\right)\right)$ of state $\rho_{L}\left(0\right)$ increases as *α* increases, and for (α=0) α=1 the state $\rho_{L}\left(0\right)$ reduces to a complete mixture state without coherence ${\mathbb{C}}_{l_{1}}\left(\rho_{L}\left(0\right)\right)=0$ (pure state with the maximum of coherence in the range of 0≤α≤1, i.e., $\max_{\alpha \in \;\left[0,1\right]}\left[{\mathbb{C}}_{l_{1}}\left(\rho_{L}\left(0\right)\right)\right]=2\sqrt{P_{00}P_{11}}$). Thus, the weight α can be regarded as an indicator to measure the coherence magnitude of $\rho_{L}\left(0\right)$. For simplicity, we, in the next subsections, will take α- indicator instead of ${\mathbb{C}}_{l_{1}}$ to measure the coherence magnitude of ancillae of CAB.

### 3.2. Thermal Modulation with Thermal Auxiliary Bath

First, we consider that the auxiliary bath is a thermal bath without coherence, i.e., each ancilla is in a thermal state, and the situation for CAB is provided in the latter subsections. Here, we focus on the modulation of heat current $J^{\mathrm{SS}}$ by the dimensionless coupling strength $\lambda_{L}$ ($\lambda_{L}=g_{L}\tau$) between the system and the ancilla with state $\rho_{L}\left(0\right)\;=\rho_{\beta }$ ($\rho_{\beta }$ given in Equation (13)) being a thermal state. For simplicity, we set the ancillae of auxiliary bath, subsystems $S_{a}$ and $S_{c}$, and the thermal TLAs of the TTB with the same transition frequencies, $\omega_{L}=\omega_{a}=\omega_{c}=\omega_{R}=\omega$ and $\omega_{b}=2\omega$, and the Planck constant ℏ=1, throughout the paper. Though some simplified treatments of parameters have been done in our model it is still hard to get the exact analytical solution of THC given in Equation (9) due to the high dimensions of system. Thus, we will investigate the features of THC numerically below when the auxiliary bath is introduced. By numerical calculations, we find that the THC $J^{\mathrm{SS}}$ behaves as a cosine-like periodical function of $\lambda_{L}$ with the fixed period $T_{\lambda }=\pi /2$, and can be fitted with the form:

(15) $${\tilde{J}}^{\mathrm{SS}}=A\cos\left(4\lambda_{L}+\varphi_{0}\right)+\Delta ,$$

where the parameters A, $\varphi_{0}$ and Δ have a complex relationship with ω, β, $\lambda_{R}$ and $\xi_{m}$ ($\xi_{m}=g_{m}\tau$ representing the coupling strength among three subsystems).

**Figure 2:**
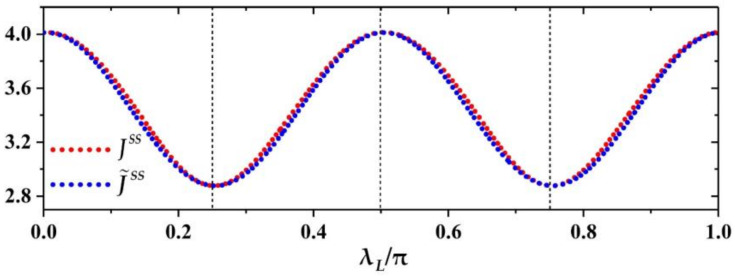
The heat current $J^{\mathrm{SS}}$ as a function of the coupling strength $\lambda_{L}$, $\lambda_{L}\in \;\left[0,\pi \right]$, in terms of Equation (9) (red dotted curve) and the corresponding curve of fitting function given in Equation (15) with A=0.567, $\varphi_{0}=0.055$ and Δ=3.445 (blue dotted curve). The other parameters are chosen as: $\lambda_{R}=0.4\pi$, $\xi_{m}=0.15\pi$, τ=0.01, ω=10 and β=0.01.

According to Equation (9) we, in Figure 2, plot the variation of THC $J^{\mathrm{SS}}$ with the coupling strength $\lambda_{L}$, $\lambda_{L}\in \;\left[0,\pi \right]$ when fixing the other parameters: $\lambda_{R}=0.4\pi$, $\xi_{m}=0.15\pi$, τ=0.01, ω=10 and β=0.01 (see the red dotted curve). Further, the blue dotted line corresponds to the fitting function ${\tilde{J}}^{\mathrm{SS}}$ with A=0.567, $\varphi_{0}=0.055$ and Δ=3.445. We can see that numerical results (red curve) of the heat current $J^{\mathrm{SS}}$ is basically matched with that of the fitting function ${\tilde{J}}^{\mathrm{SS}}$ (blue curve), i.e., $J^{\mathrm{SS}}\approx {\tilde{J}}^{\mathrm{SS}}$. Thus, the THC can be modulated in cosine-like form of $\lambda_{L}$ and satisfied as $J^{\mathrm{SS}}\left(\lambda_{L}\right){\;=J}^{\mathrm{SS}}\left(\lambda_{L}+{\mathrm{kT}}_{\lambda }\right)$ for k being zero or positive integer. The maximum (minimum) of THC is $J_{\max}^{\mathrm{Ther}}{\;=J}^{\mathrm{SS}}\left(\lambda_{L}^{\max}\right)\;=\Delta +A$ ($J_{\min}^{\mathrm{Ther}}{\;=J}^{\mathrm{SS}}\left(\lambda_{L}^{\min}\right)\;=\Delta -A$) with $\lambda_{L}^{\max}\;={\mathrm{kT}}_{\lambda }-\varphi_{0}/4≃{\mathrm{kT}}_{\lambda }$ ($\lambda_{L}^{\min}≃{\mathrm{kT}}_{\lambda }+/4$) corresponding to the minimum (maximum) strength of populations (or energy) exchange between the ancilla and the subsystem $S_{a}$ via the swap operation given in Equation (6). Physically, the nonzero steady heat current also implies that nonequilibrium steady state of system is reached. For simplicity, to denote the increment of system’s energy as ${\Delta E}_{S}$ injected by the external work source into system in M1 step of each round, the energy increment of system ${\Delta E}_{S}$ always equals to the sum of increasing amount of two baths’ energy, ${\Delta Q}_{L}+{\Delta Q}_{R}$ (the subscript L and R represent the left auxiliary bath and the right TTB, respectively), in M2 step, i.e., ${\Delta E}_{S}-\left({\Delta Q}_{L}+{\Delta Q}_{R}\right)=0$ implying no net energy accumulation in the nonequilibrium steady state dynamics. The stronger the swap strength $\left|\sin\left(2\lambda_{L}\right)\right|$ of system-ancilla (i.e., increasing or decreasing $\lambda_{L}$ in the range of $k\pi /2\leq \lambda_{L}\leq k\pi /2+\pi /4$ or $k\pi /2+\pi /4\leq \lambda_{L}\leq \left(k+1\right)\pi /2$, k=0, 1, 2, …) is, the larger the energy ${\Delta Q}_{L}$ captured by the auxiliary from the system becomes, and that results in the smaller ${\Delta Q}_{R}$ implying smaller THC $J^{\mathrm{SS}}$, vice versa. Therefore, the variety of the THC $J^{\mathrm{SS}}$ in Figure 2 with the coupling strength $\lambda_{L}$ has an opposite trend with that of swap strength, $\left|\sin\left(2\lambda_{L}\right)\right|$ with fixed period π/2. In addition, we can see that for a finite $\lambda_{L}$, $\lambda_{L}\in \;\left(0{,\;T}_{\lambda }\right)$, the THC $J_{\max}^{\mathrm{Ther}}>J^{\mathrm{SS}}\left(\lambda_{L}\neq 0\right)>J_{\min}^{\mathrm{Ther}}$ with $J_{\min}^{\mathrm{Ther}}=\Delta -A>0$ and $J_{\max}^{\mathrm{Ther}}\approx J^{\mathrm{SS}}\left(\lambda_{L}=0\right)=A\cos\varphi_{0}+\Delta$. In order to observe how the auxiliary bath influence the THC in our model, it might be appropriate take the value of THC for no interaction (i.e., $\lambda_{L}=0$) of system-ancilla as a reference THC denoted as $J_{\mathrm{ref}}$, with $J_{\mathrm{ref}}=J^{\mathrm{SS}}\left(\lambda_{L}=0\right)$. Using it, the amplification/suppression of THC can be described intuitively, that is $J^{\mathrm{SS}}>J_{\mathrm{ref}}$ and $0<J_{\mathrm{ref}}<J^{\mathrm{SS}}$ respectively correspond to the heat amplification and suppression. Here, the refrigeration is indicated by the reversal current $J^{\mathrm{SS}}<0$. Thus, Figure 2 shows that the quantum machine with the thermal auxiliary bath can only serve as a heat suppressor due to the THC being suppressed for a finite $\lambda_{L}$, and zero-current or reversal-current cannot emerge, that is, $0<J^{\mathrm{SS}}\left(\lambda_{L}\neq 0\right){<J}_{\mathrm{ref}}$. Meanwhile, by changing the coupling strength $\lambda_{L}$ the thermal suppressor can modulate THC in the form of cosine-like function in the range of $\Delta +A\geq J^{\mathrm{SS}}\geq \Delta -A$.

Here, in order to conveniently describe the capability of quantum machine to modulate the THC by adjusting the controllable coupling strength, we define modulation width of the quantum machine on THC as the difference of the maximum and the minimum of THC, i.e.,

(16) $$D\;:=J_{\max}^{\mathrm{SS}}\left(\lambda_{L}^{}\right)=J_{\min}^{\mathrm{SS}}\left(\lambda_{L}^{}\right)$$

with $0\leq \lambda_{L}\leq T_{\lambda }$. As for a thermal control device, one usually expects that it could control the THC varying over a wide range as much as possible. That is, the larger the modulation width of THC is, the better the performance of quantum machine is. In terms of characteristics of THC given in Equation (15), the system with the thermal auxiliary bath can only work as a thermal suppressor with the modulation width D=2A.

### 3.3. Thermal Modulation with CAB

We, in this subsection, mainly focus on the behaviors of THC and the thermal functions of quantum machine assisted with a CAB.

#### 3.3.1. Effects of Relative Phase on THC

**Figure 3:**
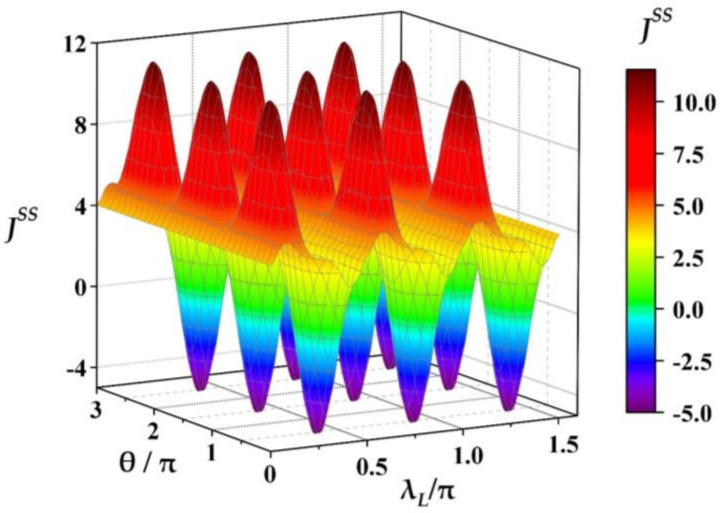
The THC $J^{\mathrm{SS}}$ as a periodical function of θ and $\lambda_{L}$ with $\theta \in \;\left[0,\;3\pi \right]$ and $\lambda_{L}\in \;\left[0,\;0.15\pi \right]$. The other parameters are set as: α=1, $\lambda_{R}=0.4\pi$, $\xi_{m}=0.15\pi$, τ=0.01, ω=10 and β=0.01.

**Figure 4:**
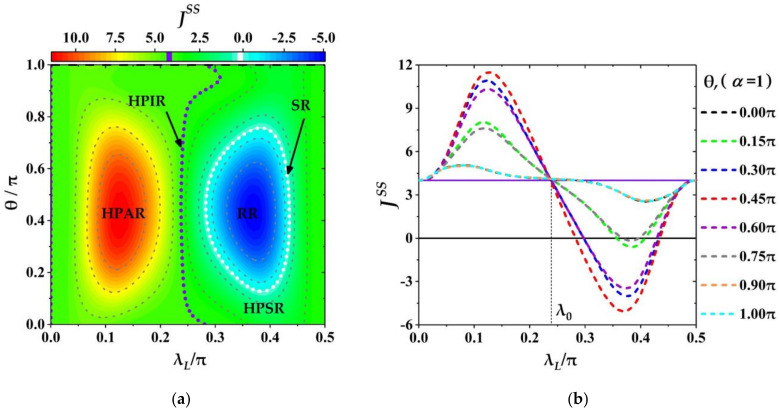
The variations of THC $J^{\mathrm{SS}}$ as θ and $\lambda_{L}$. (**a**) The phase diagram of the quantum machine working as a multifunctional device in the parametric regimes of θ and $\lambda_{L}$: RR, SR, HPSR, HPIR and HPAR; (**b**) the variations of $J^{\mathrm{SS}}$ with $\lambda_{L}$ for some fixed relative phases θ. The other parameters are the same as the ones in Figure 3. The purple (white) dotted line in (**a**) represents working points with $J^{\mathrm{SS}}=J_{\mathrm{ref}}$ ($J^{\mathrm{SS}}=0$) also corresponding to the purple (black) solid line in (**b**).

Next, we study the effects of the relative phase θ and the coupling strength $\lambda_{L}$ of ancillae on the THC $J^{\mathrm{SS}}$ numerically for a fixed magnitude of coherence below. From numerical calculations we find that when the thermal auxiliary bath is replaced by the CAB the THC $J^{\mathrm{SS}}$ is a periodical function of $\lambda_{L}$ and θ, i.e., $J^{\mathrm{SS}}\left(\lambda_{L}+T_{\lambda },\;\theta +T_{\theta }\right){=J}_{\mathrm{ref}}\left(\lambda ,\;\theta \right)$ with $T_{\lambda }=\pi /2$ and $T_{\theta }=\pi$ as shown in Figure 3, where each ancilla of CAB is prepared in the same state, $\rho_{L}\left(0\right)=|\phi_{L}\rangle \langle \phi_{L}|$ given in Equation (13) with the maximum magnitude of coherence α=1. It is noted that the periods $T_{\lambda }=\pi /2$ and $T_{\theta }=\pi$ are independent of the other parameters in our model. In order to demonstrate the characteristics of $J^{\mathrm{SS}}$ and the thermal functions of quantum machine clearly, the variation of $J^{\mathrm{SS}}$ in a single period with $0\leq \theta \leq T_{\theta }$ and $0\leq \lambda_{L}\leq T_{\lambda }$ is shown in Figure 4 where all the other parameters are the same as that given in Figure 3. In Figure 4a the multifunctional regions of quantum machine have been shown, and Figure 4b for the corresponding variations of $J^{\mathrm{SS}}$ for some fixed θ, $\theta /\pi =\left\{0,\;0.15\right.,\;0.30$ $\left.0.45,\;0.60,\;0.75,\;0.90,\;1.0\right\}$. From Figure 4a it can be seen that the quantum machine could work as a multifunctional thermal device, and the specific function relies on the values of parameters θ and $\lambda_{L}$. Specifically, in terms of the features of $J^{\mathrm{SS}}$ in Figure 4a, the whole parametric space of $0\leq \lambda_{\theta }\leq T_{\theta }$ and $0\leq \theta \leq T_{\theta }$ is divided into several different function regions: switcher region (SR) (the white dotted line for $J^{\mathrm{SS}}=0$), refrigeration region (RR) (the region surrounded by the white dotted line for $J^{\mathrm{SS}}<0$), heat pump invariable region (HPIR) (the purple dotted line for $J^{\mathrm{SS}}=J_{\mathrm{ref}}$), heat pump suppression region (HPSR) (the middle region between the white and purple dotted lines for $J_{\mathrm{ref}}>J^{\mathrm{SS}}>0$) and heat pump amplification region (HPAR) (the left-side region of purple line for $J^{\mathrm{SS}}>J_{\mathrm{ref}}$).

In addition, Figure 4a also demonstrates several obvious features. First, each functional region of HPAR, HPIR, HPSR, SR and RR distributes in a certain continuous parametric space of $\lambda_{L}$ and θ. Second, for the convenient descriptions of different functional regions we denote $\lambda_{u}$ as the coupling strength of HPIR (the purple dotted line at the middle region in Figure 4a), satisfying $J^{\mathrm{SS}}\left({\theta ,\;\lambda }_{u}\right)=J^{\mathrm{SS}}\left({\theta ,\;\lambda }_{L}=0\right)=J_{\mathrm{ref}}$ (note that $J^{\mathrm{SS}}\left({\theta ,\;\lambda }_{u}\right)=J_{\mathrm{ref}}$ does not mean the redefinition of the reference current $J_{\mathrm{ref}}$, and only indicates the values of THC at some certain working points $\left({\theta ,\;\lambda }_{u}\right)$ on the purple dotted line are the same as reference current $J_{\mathrm{ref}}\left(\lambda_{L}=0\right)$ as defined before). One can see that the HPAR only lies in the region of $\lambda_{L}$ below $\lambda_{u}$, (i.e., the region of $\lambda_{L}<\lambda_{u}$), and other function regions of HPSR, SR and RR for $\lambda_{L}$ over $\lambda_{u}$, (i.e., the region of $\lambda_{L}>\lambda_{u}$). Further, the SR and RR appear in the middle region of parametric space: $\theta \in \;\left[{0,\;T}_{\theta }\right]$ and $\lambda_{L}\in \;\left[\lambda_{u}{,\;T}_{\lambda }\right]$, and the reminder part for the HPSR. Third, for the relative phase θ taken in RR the quantum machine could perform the different thermal functions in turn: thermal amplifier→stabilization→suppressor→switcher→refrigerator→suppressor as $\lambda_{L}$ increases from zero to the maximum $T_{\lambda }$ in a period.

From Figure 4b, one can see some specific behaviors of $J^{\mathrm{SS}}$ varying with θ and $\lambda_{L}$. Firstly, for a finite relative phase θ the THC $J^{\mathrm{SS}}$ always behaves as a sine-like function with respect to $\lambda_{L}$. That is, the THC $J^{\mathrm{SS}}$ first increases from the initial value $J_{\mathrm{ref}}$ to the maximum then decreases to the minimum below $J_{\mathrm{ref}}$, and monotonically returns to its initial value again. Secondly, for the coupling strength $\lambda_{L}$ being about 0.24π denoted as $\lambda_{0}=0.24\pi$ the values of $J^{\mathrm{SS}}$ with different θ approach to the same value of $J_{\mathrm{ref}}$, i.e., $J^{\mathrm{SS}}\left({\theta ,\;\lambda }_{u}\right)\approx J_{\mathrm{ref}}$ which means that for the critical coupling strength $\lambda_{0}=0.24\pi$ the THC is almost independent of the relative phase. Thirdly, in the region of $J^{\mathrm{SS}}>J_{\mathrm{ref}}$ (HPAR) and fixing $\lambda_{L}$ the closer the relative phase θ is about to 0.45π denoted as $\theta_{S}=0.45\pi$, the larger the THC $J^{\mathrm{SS}}$ becomes (except for the larger θ such as θ/π=0.75, 0.9, 1.0 where the three curves of $J^{\mathrm{SS}}$ are basically overlap, i.e., the less influence the larger relative phases have), but for the region of $J^{\mathrm{SS}}<J_{\mathrm{ref}}$ the opposite is true (where $J^{\mathrm{SS}}$ decreases as θ closes to $\theta_{S}$, and it can be less than or equal to zero seen the segments below the black solid line with $J^{\mathrm{SS}}=0$).

Based on the numerical simulations above, we show that the relative phase of ancillae is related to the THC and could be regarded as a useful resource to modulate the THC well. Meanwhile, at the suitable relative phase of ancillae the quantum machine can integrate multiple functions, such as thermal amplifier, stabilizer, suppressor, switcher and refrigerator, and these functions can be switched only by adjusting the coupling of system-CAB.

#### 3.3.2. Effects of Coherence Magnitude on THC

Next, we mainly concern the influences of coherence magnitude α on the THC $J^{\mathrm{SS}}$ for a fixed relative phase $\theta_{S}$ (here, $\theta_{S}=0.45\pi$ corresponds to the largest modulation width of $J^{\mathrm{SS}}$ (seen in Figure 4a or Figure 4b), i.e., $\max\left[D\left(\lambda_{L},\;\theta \right)\right]=D\left(\lambda_{L}{,\;\theta }_{S}\right)$ with $0\leq \theta_{S}\leq \pi$, where $D\left(\lambda_{L},\;\theta \right)$ has been given in Equation(16)).

**Figure 5:**
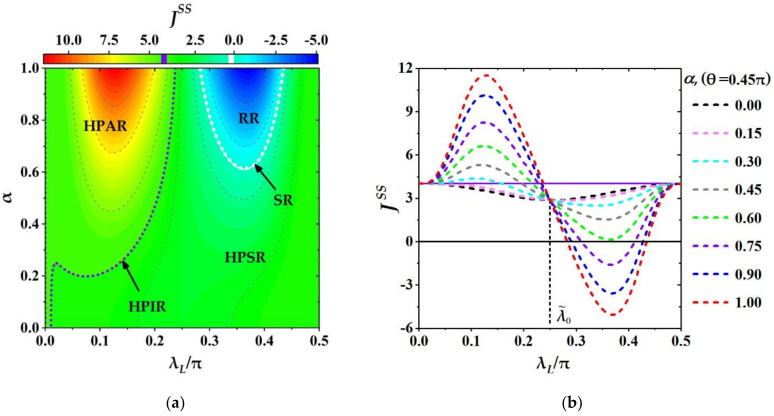
The variation of $J^{\mathrm{SS}}$ with α and $\lambda_{L}$. (**a**) The phase diagram of the quantum machine behaving as a multifunctional device in parameter space: 0≤α≤1 and $0\leq \lambda_{L}\leq \pi /2$; (**b**) the THC $J^{\mathrm{SS}}$ as function of $\lambda_{L}$ for some selected α. The other parameters are chosen by $\lambda_{R}=0.4\pi$, $\xi_{m}=0.15\pi$, τ=0.01, ω=10 and β=0.01.

In Figure 5a, we show the different function regions in the parametric space of $0\leq \lambda_{L}\leq \pi /2$ and 0≤α≤1, including: SR (the white dotted line for $J^{\mathrm{SS}}=0$), HPIR (the purple dotted line for $J^{\mathrm{SS}}\left(\lambda_{0},\;\alpha \right)=J_{\mathrm{ref}}$), HPAR (the left part of the purple dotted line for $J^{\mathrm{SS}}\left(\lambda_{L},\;\alpha \right)>J_{\mathrm{ref}}$), HPSR (the middle regime between the purple and the white dotted lines for $0<J^{\mathrm{SS}}\left(\lambda_{L},\;\alpha \right)<J_{\mathrm{ref}}$) and RR (the space surrounded by the white dotted line for $J^{\mathrm{SS}}<0$). From Figure 5a it can be seen that for small coherence magnitude α (about α<0.2) the HPAR with $\lambda_{L}<\lambda_{0}$ becomes very narrow (i.e., for α<0.2 the coupling strength $\lambda_{0}$ in HPIR is also small), and the rest region of $\lambda_{L}$ is for the HPSR with $\lambda_{L}<\lambda_{0}$. It means that for the small *α* the quantum machine could serve as a thermal amplifier in the regime of $\lambda_{L}<\lambda_{0}$, and the thermal suppressor in the regime of $\lambda_{L}{>\lambda }_{0}$. However, as α increases the domain of HPAR/HPSR enlarges/shrinks rapidly, and when α is beyond a certain value (about 0.6 with $\min\left[J^{\mathrm{SS}}\left(\lambda_{0},\;\alpha =0.6\right)\right]\approx 0$ seen the Figure 5b, the SR and RR can appear in the regime $\lambda_{L}{>\lambda }_{0}$, and both of SR and RR enlarge as α increases.

To demonstrate the dependence of $J^{\mathrm{SS}}$ on α and $\lambda_{L}$ clearly, we, in Figure 5b, plot the variations of $J^{\mathrm{SS}}$ with respect to $\lambda_{L}$ for some selected α, $\alpha =\left\{0,\;0.15,\;0.3,\;0.45,\;\right.$$\left.0.6,\;0.75,\;0.9,\;1.0\right\}$. Further, some specific features of $J^{\mathrm{SS}}$ have been shown in Figure 5b. Firstly, for a fixed α the THC $J^{\mathrm{SS}}$ always rises at first, then falls down, and rises up its initial value again. Meanwhile, the modulation width $D\left(\lambda_{L},\;\alpha \right)$ of the THC becomes larger with the increase of α. Secondly, we notice that for $\lambda_{L}={\tilde{\lambda }}_{0}$ with ${\tilde{\lambda }}_{0}=0.25\pi$, the THCs with different α have the same value as the one for thermal auxiliary bath (α=0), i.e., $J^{\mathrm{SS}}\left({\tilde{\lambda }}_{0},\;\alpha \right)=J^{\mathrm{SS}}\left({\tilde{\lambda }}_{0},\;0\right)$ with $\alpha \in \;\left[0,\;1\right]$, which means that at the critical coupling $\lambda_{L}={\tilde{\lambda }}_{0}$ the effects of coherence on THC can be frozen, i.e., the THC is independent of the coherence magnitude of ancillae. For $0<\lambda_{L}<{\tilde{\lambda }}_{0}$ the THC can be amplified by the coherence of CAB, i.e., $J^{\mathrm{SS}}\left(\lambda_{L},\;\alpha \right)>J^{\mathrm{SS}}\left(\lambda_{L},\;0\right)$ (seen for $0<\lambda_{L}<{\tilde{\lambda }}_{0}$ all the lines of $J^{\mathrm{SS}}$ with different non-zero α are above the black solid line with α=0), and for a fixed $\lambda_{L}$ the THC $J^{\mathrm{SS}}$ always increases monotonously with the increasing of coherence magnitude α, which implies that the THC is positively correlated with the coherence magnitude of ancillae. However, for $\lambda_{L}>{\tilde{\lambda }}_{0}$ the situation is opposite where the THC $J^{\mathrm{SS}}$ is suppressed, $J^{\mathrm{SS}}\left(\lambda_{L},\;\alpha \right)=J^{\mathrm{SS}}\left(\lambda_{L},\;0\right)$ (seen all the lines with different α are below the one with α=0 for $\lambda_{L}>{\tilde{\lambda }}_{0}$), and the value of $J^{\mathrm{SS}}\left(\lambda_{L},\;\alpha \right)$ with fixed $\lambda_{L}$ decreases monotonously with increasing α, which demonstrates that the THC is negatively correlated with the coherence magnitude of ancillae in the regime of $\lambda_{L}>{\tilde{\lambda }}_{0}$. Especially, for the ancillae with strong coherence (α>0.6) the zero- or reversal-current, $J^{\mathrm{SS}}\leq 0$, can appear (each curve of $J^{\mathrm{SS}}$ with different α, (α=0.75, 0.9, 1.0), has one segment below the black solid line with $J^{\mathrm{SS}}=0$) which means that for the CAB with strong coherence the quantum machine can also serve as a switcher or a refrigerator.

Based on the analysis above, one can find that the THC is related to not only the coupling of system-ancillae but also the coherence magnitude of ancillae. Meanwhile, the influences of coherence magnitude of ancillae on the THC, such as amplification, suppression and reverse of THC, strongly depend on the coupling strength of system-ancillae. This can be understood that when thermal auxiliary bath is replaced with the CAB the system will reach a new steady state associated with the ancilla’s coherence magnitude and relative phase. Based on some previous researches on the thermalization problem including the thermalization of the TLA/micro-cavity as system by a coherent TLAs/three-level atoms (or atomic-pairs) bath (seen in [94,95,96]) it is known that except for the population of bath units (the coherent TLAs/three-level atoms (or atomic-pairs)) the coherence in bath units and the coupling strength between the system and the units have a nonlinear effect on the coherence and the population of system, and the respective contributions of the coherence (coherence magnitude and relative phase) and the coupling strength to the population of system at steady state cannot be separated though the coherence and the coupling strength are independent parameters. Meanwhile, compared with the thermal bath without coherence the excite populations of system at steady state cannot only increase but also decrease which is determined by the parameters of coherence and coupling strength together. This might be why the THC can exhibit rich behaviours when the TTB is introduced. Thus, based on the characteristics of THC one can see that for a prepared CAB the THC can be modulated well by adjusting the controllable coupling of system-ancilla. Especially, for the ancillae with strong coherence (large α) the THC can go through all the regions: HPAR, HPIR, HPSR, SR and RR orderly only by increasing the coupling strength $\lambda_{L}$ from zero to $T_{\lambda }$.

#### 3.3.3. Maximum and Minimum of THC and Modulation Width

We have shown the characteristics of THC and the thermal functions of quantum machine at fixing coherence magnitude α=1 (or relative phase θ=0.45π) before. It demonstrates that quantum machine could behave the different functions in different parametric space of $\lambda_{L}$, α and θ. Now, we observe which thermal functions the machine can perform when the coherence parameters, α and θ (0≤α≤1 and 0≤θ≤π), of ancillae are arbitrary.

**Figure 6:**
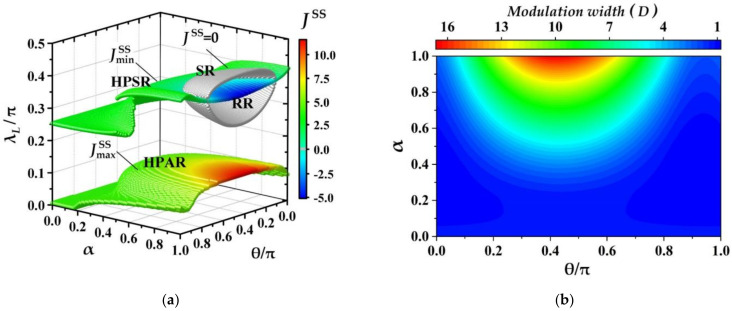
(**a**) The maximum and the minimum of THC, $J_{\max}^{\mathrm{SS}}$ and $J_{\min}^{\mathrm{SS}}$, and function regions of quantum machine; (**b**) the modulation width of THC, $D=J_{\max}^{\mathrm{SS}}-J_{\min}^{\mathrm{SS}}$, in in the full parametric space: $\lambda_{L}\in \;\left[0,\;\pi /2\right]$, $\alpha \in \;\left[0,\;1\right]$ and $\theta \in \;\left[0,\;\pi \right]$. The other parameters are set as $\lambda_{R}=0.4\pi$, $\xi_{m}=0.15\pi$, τ=0.01, ω=10 and β=0.01.

Due to the continuous variation of THC with the parameters $\lambda_{L}$, α and θ, the thermal functions of quantum machine are determined by the maximum $J_{\max}^{\mathrm{SS}}$ and the minimum $J_{\min}^{\mathrm{SS}}$ of THC, i.e., to judge which functional regions of HPAR, HPIR, HPSR, SR and RR are covered in the range of $J_{\min}^{\mathrm{SS}}$~$J_{\max}^{\mathrm{SS}}$. Therefore, we plot the maximum and the minimum of THCs, $J_{\max}^{\mathrm{SS}}$ and $J_{\max}^{\mathrm{SS}}$, in Figure 6a and the corresponding modulation width of THC, $D=J_{\max}^{\mathrm{SS}}-J_{\min}^{\mathrm{SS}}$, in Figure 6b in the full parametric space of $\lambda_{L}$, α and θ with $0\leq \lambda_{L}\leq T_{\lambda }$, 0≤α≤1 and 0≤θ≤π. The lower and upper colorful surfaces in Figure 6a correspond to the variations of $J_{\max}^{\mathrm{SS}}$ and $J_{\min}^{\mathrm{SS}}$, respectively, and the sliver gray ellipsoid-like surface for the working points of switcher with $J^{\mathrm{SS}}=0$. It is noticed that the values of $J_{\max,\min}^{\mathrm{SS}}$ are characterized by the colors on surfaces not the height of the surfaces (the height of surface is for the vertical axis $\lambda_{L}$). From Figure 6a, several features of $J_{\max}^{\mathrm{SS}}$ and $J_{\min}^{\mathrm{SS}}$ have been shown. Firstly, it can be seen that the surface of $J_{\max}^{\mathrm{SS}}$ is always above the one of $J_{\min}^{\mathrm{SS}}$ which indicates that for the fixed coherence parameters of (α, θ) the maximum of THC $J_{\max}^{\mathrm{SS}}$ corresponds to the small $\lambda_{L}$ being about $0<\lambda_{L}<0.15\pi$, and the minimum of THC $J_{\min}^{\mathrm{SS}}$ for the large $\lambda_{L}$ with $0.25\pi <\lambda_{L}<0.45\pi$. Secondly, $J_{\max}^{\mathrm{SS}}$ and $J_{\min}^{\mathrm{SS}}$ behave as the positive and the negative correlated to the coherence magnitude *α*, respectively, i.e., the value of $J_{\max}^{\mathrm{SS}}$ ($J_{\min}^{\mathrm{SS}}$) increases (decreases) as α increases, which implies that the modulation width, $D=J_{\max}^{\mathrm{SS}}-J_{\min}^{\mathrm{SS}}$, of THC is an increasing function of α as shown in Figure 6b. Thirdly, the maximum (minimum) values of THC are always larger (smaller) than the reference THC, i.e., $J_{\max}^{\mathrm{SS}}>J_{\mathrm{ref}}$ ($J_{\min}^{\mathrm{SS}}{<J}_{\mathrm{ref}}$) with $J_{\mathrm{ref}}=J^{\mathrm{SS}}\left(\lambda_{L}=0,\;\alpha =0\right)$. Therefore, $J_{\max}^{\mathrm{SS}}$ always remains in HPAR, and $J_{\min}^{\mathrm{SS}}$ could distribute into three regions: HPSR ($0<J_{\min}^{\mathrm{SS}}<J_{\mathrm{ref}}$), RR ($J_{\min}^{\mathrm{SS}}=0$) corresponding the parts outside and inside the gray ellipsoid-like surface, and SR ($J_{\min}^{\mathrm{SS}}=0$). In addition, from Figure 6b one can see that the larger of the coherence magnitude α and the closer to relative phase θ is to 0.45π, the wider the modulation width of THC becomes, and the better the performance of quantum machine is.

With respect to the characteristics of $J_{\max,\min}^{\mathrm{SS}}$ above, one can infer that the quantum machine assisted with CAB can always work as a heat amplifier or a suppressor by tuning the coupling strength $\lambda_{L}$, $\lambda_{L}\in \;\left[0,\;\pi /2\right]$, due to both regions of HPAR and HPSR being covered in the full parametric space, (α, θ), of coherence. Meanwhile, for some regimes of (α, θ) with $J_{\min}^{\mathrm{SS}}=0$ (or $J_{\min}^{\mathrm{SS}}<0$) the quantum machine can also work as a switcher (a switcher or a refrigerator) due to the SR (or RR) being involved. Especially, in full coherent parametric space of 0<α≤1 and 0≤θ≤π the maximum (minimum) of $J_{\max}^{\mathrm{SS}}$ ($J_{\min}^{\mathrm{SS}}$) exists at α=1 and θ=0.45π, i.e., $\max\left[J_{\max}^{\mathrm{SS}}\left(\alpha ,\;\theta \right)\right]=J_{\max}^{\mathrm{SS}}\left(1,\;0.45\pi \right)$ ($\min\left[J_{\min}^{\mathrm{SS}}\left(\alpha ,\;\theta \right)\right]=J_{\min}^{\mathrm{SS}}\left(1,\;0.45\pi \right)<0$) which also corresponds to the largest modulation width of THC (seen Figure 6b). Thus, the CAB consisting of the ancillae with α=1 and θ=0.45π can be regarded as the optimal CAB, in which the quantum machine not only integrates all thermal functions as a heat amplifier, suppressor, switcher and refrigerator, but also could perform the strongest capabilities in heat amplification and refrigeration.

#### 3.3.4. Effect of Temperature on THC

**Figure 7:**
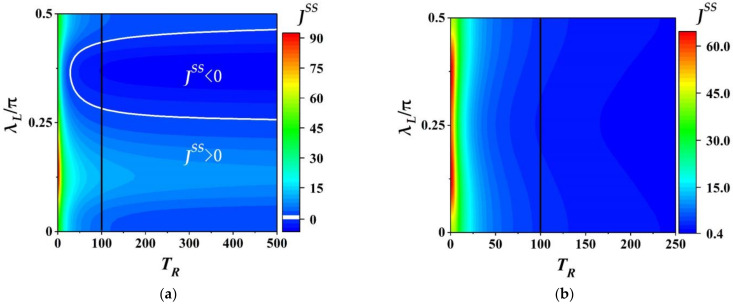
The heat current $J^{\mathrm{SS}}$ as a function of the temperature of TTB $T_{R}$ and the coupling strength $\lambda_{L}$ with $\lambda_{L}\in \;\left[0,\;0.5\pi \right]$ for the CAB with α=1 and θ=0.45π in (**a**), and the thermal auxiliary bath in (**b**). Here, the white solid line in (a) represents working points with $J^{\mathrm{SS}}=0$, and the black solid line in (a,b) indicate the position with equal temperatures $T_{L}=T_{R}=100$. The other parameters: $\lambda_{R}=0.4\pi$, $\xi_{m}=0.15\pi$, τ=0.01, ω=10 and β=0.01.

From numerical simulation we investigate the effects of temperature on THC. We, in Figure 7, plot the variation of target heat current (THC) $J^{\mathrm{SS}}$ with the temperature of TTB, $T_{R}$, and the coupling strength, $\lambda_{L}$, for fixed $\beta_{L}=0.01$. Further, Figure 7a,b respectively depict the behaviors of THC for the auxiliary being the CAB and the thermal auxiliary bath. From Figure 7a one can see that two obvious features of THC $J^{\mathrm{SS}}$ have been shown. First, for the fixed $\lambda_{L}$ the THC $J^{\mathrm{SS}}$ always decreases as the temperature $T_{R}$ increases, and approaches to the steady value for the high enough $T_{R}$. Second, for different $T_{R}$ features of the THC $J^{\mathrm{SS}}$ varying with $\lambda_{L}$ are similar. However, for different $\lambda_{L}$ the THC $J^{\mathrm{SS}}$ can exhibit different behaviors as $T_{R}$ increases. For example, in the range of $0.25\pi \leq \lambda_{L}\leq 0.47\pi$ the THC $J^{\mathrm{SS}}$ always decreases from a relative large positive value to zero, and then increases in the opposite direction. This means that the quantum machine in this coupling region can perform a heat pump ($J^{\mathrm{SS}}>0$ region outside the white curve), switching ($J^{\mathrm{SS}}=0$ the working points on the white curve) or refrigerator ($J^{\mathrm{SS}}<0$ region inside the white curve) which depends on the temperature of TTB. It is noted that for the coupling strength about $\lambda_{L}\leq 0.25\pi$ and $0.47\pi \leq \lambda_{L}\leq 0.5\pi$ the heat always flow into the TTB $J^{\mathrm{SS}}>0$ no matter $T_{R}$ is higher or lower than $T_{L}$. Thus, the machine assisted with the coherent auxiliary bath can also exhibit thermal-diode-like action in the certain parametric regimes. Further, the amplification (refrigeration) of the machine with CAB can be enhanced in the heating (cooling) region with $T_{R}<T_{L}$ (the left side of black solid line) ($T_{R}>T_{L}$ (the right side of black solid line)). That is, the different temperatures could boost the performance of the machine in the certain region of $\lambda_{L}$.

Compared with the situation of CAB for the thermal auxiliary bath shown in Figure 7b, though the THC also decreases as the temperature $T_{R}$ it cannot be less than or equal to zero in whole parametric space of $0\leq \lambda_{L}\leq 0.5\pi$ and $0\leq T_{R}\leq 250$, which implies that the device assisted with the thermal auxiliary bath cannot work as a thermal switch or refrigerator.

## 4. Conclusions

In summary, we have proposed a scheme of heat modulation via a three-partite system assisted with a CAB to control the magnitude and the direction of heat current between the system and the TTB. We have analyzed the influences of quantum coherence of ancillae, the coupling strength of system-ancillae and the temperature of two baths on the THC at length. It is shown that for the thermal auxiliary without coherence, the THC behaves as a cosine-like variation with the coupling strength of system-ancillae and a suitable fitting function has been given. Meanwhile, under the thermal auxiliary bath, no matter the high or the low temperature of TTB the heat always flows into the TTB implying the device assisted with the thermal auxiliary bath only working as a heat pump. However, replacing the thermal auxiliary bath with the CAB, due to the influence of coherence (including the coherence magnitude of and the relative phase) of ancillae the THC, in certain parametric regimes of coherence and the coupling of system-ancillae, could exhibit rich behaviors, such as heat amplification, heat suppression, zero- and reversal-current. Therefore, the three-partite system assisted with the CAB could serve as a multifunctional thermal device integrating with heat amplifier, suppressor, switcher and refrigerator. Via the analysis of the maximum and the minimum THC in full coherence parametric space of coherence magnitude and relative phase, the optimal CAB can be suggested, in which the modulation width of THC is largest, and the machine could perform the strongest capabilities in heat amplification and refrigeration. Besides, it has been demonstrated that the different thermal functions can be switched flexibly only by adjusting the coupling strength of system-ancillae, which is convenient for practical application.

Our research might shed some light on the role of resource of quantum coherence outside the system, could boost the deep understanding of thermodynamic properties of quantum coherence, and provide a new perspective for the design of multifunctional thermal management device with the aid of a non-equilibrium auxiliary bath.

## Data Availability

Data sharing not applicable.
